# Device-tissue interactions: a collaborative communications system

**DOI:** 10.1186/1750-1164-7-10

**Published:** 2013-07-29

**Authors:** Edward Chekan, Richard L Whelan, Alexander H Feng

**Affiliations:** 1 Ethicon Endo-Surgery, Inc. (EC), 4545 Creek Road, Cincinnati, OH, 45242, USA; 2 St. Luke’s Roosevelt Hospital (RLW), New York, NY, USA; 3 the dd+p group, Cincinnati, OH, USA

**Keywords:** Access-enabling device, Education, Energy device, Patient outcome, Stapler, Surgeon, Tissue

## Abstract

Medical devices, including surgical staplers, energy-based devices, and access enabling devices, are used routinely today in the majority of surgical procedures. Although these technically advanced devices have proved to be of immense benefit to both surgeons and patients, their rapid development and continuous improvement have had the unintended consequence of creating a knowledge gap for surgeons due to a lack of adequate training and educational programs. Thus, there is an unmet need in the surgical community to collect existing data on device-tissue interactions and subsequently develop research and educational programs to fill this gap in surgical training. Gathering data and developing these new programs will require collaboration between doctors, engineers, and scientists, from both clinical practice and industry. This paper presents a communications system to enable this unique collaboration that can potentially result in significantly improved patient care.

## Introduction

Technological advancements in the highly specialized field of operative surgical management have been occurring at an unprecedented pace. More specifically, the continuous introduction of novel devices and instruments are changing the way surgeons perform many tasks, and, in some cases, creating entirely new surgical techniques and possibilities [1-6]. This is particularly evident when new devices are combined with previously existing therapeutic modalities. At the same time, the scientific basis for the optimal use of these new devices has frequently not been established, and there is often an educational gap in surgeons’ understanding of modern tissue management [7]. A fundamental scientific understanding of how these surgical devices interact with tissues should be a universal requirement for all surgeons [8,9]. As a first step in this process, the existing data on the topic, as known to surgeons and as available in literature, should be collected, collated, and organized into a simple structure as recommended below, to be expanded upon with information made available by manufacturers. A systematic research program can then follow, and educational programs to fill this gap in surgical training should be developed. Such programs will require collaboration between doctors, engineers, and scientists, from both clinical practice and industry.

A panel discussion at a recent Society of American Gastrointestinal and Endoscopic Surgeons (SAGES) Annual Meeting was held to explore surgery device and tissue interactions [10]. During this discussion, the authors specifically addressed the use of stapling and energy devices, and frequently polled the audience to assess their understanding of how these devices and tissues interact. Numerous questions were asked by the panel, including queries such as “Do you routinely oversew staple lines?” and “How do you choose a staple cartridge during a gastric bypass procedure?” Though unscientific, the collective polling results of the evening were revealing, in that they brought to light a knowledge gap in surgeons’ understanding of the interaction between modern tissue management devices and tissue, indicating a necessity for additional education. After the SAGES event, we agreed to collaborate in an effort to help bridge this knowledge gap: the first necessary steps were (1) to agree on a framework to house the available information regarding device-tissue interaction; and (2) to jointly publish a paper calling for the community of surgeons, researchers, and device makers to provide information in the framework in order to improve patient outcomes.

Achieving optimal patient outcomes is the goal of every surgeon. The successful outcome of a surgical problem ultimately involves many layers of interactions, including interacting with third party payers, the patients and their families. At the deepest level, there is the complex interaction of numerous devices with tissue. Here, there is a complex web of technologies that must be managed in order to achieve the desired overall therapeutic result. Moreover, during the moments in which each device is applied to a specific piece of tissue, there is a specific result for each of these actions. These are the actions that we are describing as the device/tissue interaction.

The purpose of this paper is to define the framework for discussion around the device/tissue interaction in order to organize the scientific literature that is currently available on the topic. A framework, or common ground, will help to drive conversation, collaboration, and innovation, and ultimately result in positive/optimal patient outcomes. In addition, it will serve to point out areas requiring further study.

### Device-tissue interactions – a framework

#### Device

An unwieldy list of devices and device characteristics can be created if all devices used in the operating room are considered (e.g., staplers, cautery instruments, trocars, mesh, sponges, scissors, scalpels, hand instruments, cameras, implants, bags, manipulators, and many more, not to mention those instruments which do not have direct patient contact). Consequently, in order to address the most immediate educational gaps first, this paper will focus on those device groups that are considered to be: (1) critical (used in important operative tasks); (2) compound (used for more than one operative job and across most operative procedures); and (3) complex (comprised of many interconnecting components).

To this end, three specific device categories familiar to most surgeons can be used as examples: namely, stapling, energy-based, and access-enabling devices. Stapling devices are defined as those instruments designed to simultaneously appose two edges of tissue and deliver a line of staples, thus securing the tissue apposition and, in turn, promoting healing (these devices may also transect tissue as part of their function). Energy devices are those devices that are electrically activated and serve to transfer electrical, or ultrasonic, energy from the device to the tissue; their function is to cut and/or coagulate tissue in surgery. Finally, access-enabling devices are designed to provide access to the specific operative space (e.g., abdominal, thoracic) and adequate domain to perform necessary tissue manipulation while minimizing tissue trauma.

Although stapling, energy-based, and access-enabling devices are designed to perform distinctly different functions, there exist certain knowledge elements common to each category that when utilized in practice can help to achieve an optimal patient outcome, irrespective of the specific device. However, a lack of awareness of these elements by many surgeons constitutes part of the previously described knowledge gap that needs to be addressed. In the simplest form, these common elements are as follows: knowing what type of unit the device uses, understanding the intended outcome of the device, and knowing which type of device embodies that function. (See Additional file 1: Table S1 for this list).

#### Tissue

A wide variety of tissues are encountered in every operative procedure and the characteristics of these tissues can vary based on a patient’s co-morbid conditions. Categories that help to differentiate tissue include intrinsic tissue properties and extrinsic blood supply (i.e., perfusion). Tissue properties can then be broken down into mechanical (i.e., liquid versus air versus solid components) and biochemical properties (i.e., protein content, metabolic profile). Manipulating adequately perfused tissue can be expected to have a different outcome versus poorly perfused tissue.

#### Device-tissue interaction

Finally, there is the dynamic and complex process that occurs when devices and tissues interact. The goal of this interaction is to achieve the desired patient outcome through the gentle and precise manipulation of various types of tissue, which will reduce tissue injury and promote healing. Consideration of this kinematic involvement by itself adds another layer of knowledge that is an opportunity for exploration– for example, the resultant tissue compression or tension during the use of stapling devices [11] or resultant heat and/or motion for energy devices during surgery.

A more complete list of questions and knowledge areas for each of the areas described above are presented in Additional file 2: Table S2, Additional file 3: Table S3 and Additional file 4: Table S4.

By exploring these areas in detail, clinicians, biologists, and engineers can contribute to better patient care by expanding the collective knowledge base of device-tissue interactions. Indeed, we believe this endeavor requires both clinicians and industry working together in concert, as neither has appropriate resources when working in isolation. When surgeons and industry pool their respective knowledge and experience, a successful device can be created that will be more effective for the surgeon and benefit patients by improving clinical outcomes. This endeavor represents an opportunity for a unique collaboration that can potentially result in significantly improved patient care.

## Conclusions

The technology of operative surgical management is rapidly evolving, with the introduction of devices and instruments that are changing the way surgeons perform many tasks, as well as creating novel techniques and new possibilities. Unfortunately, the scientific foundation and protocols for the optimal use of these technologically advanced devices has not been established. Moreover, there is an educational gap in surgeons’ understanding of modern tissue management. A fundamental scientific understanding of how devices interact with tissues is required. The existing data on the topic, as known to surgeons and as available in literature, should be collected, collated, and organized into a simple structure as recommended here, to be expanded upon with information made available by manufacturers. The data-organizing phase should set the stage for systematic research programs (the results of which can be published and added to this structure), followed by the development of educational programs to fill this gap in surgical training. Development of these important and necessary programs will require collaboration between doctors, engineers, and scientists, from both clinical practice and industry, and would serve to tabulate and summarize the collected information, with a goal of periodic evidence summaries to be released on a biannual basis.

## Competing interests

Edward Chekan, MD, is an employee of Ethicon Endo-Surgery and holds stock in Johnson & Johnson. Richard L. Whelan, MD, serves as a consultant for Ethicon Endo-Surgery. Alexander H. Feng serves as a consultant for Ethicon Endo-Surgery.

## Authors’ contributions

EC, MD, participated in creating, drafting, editing, and reviewing the manuscript. RLW, MD, participated in editing and reviewing the manuscript. AHF participated in creating, drafting, editing, and reviewing the manuscript. All authors read and approved the final manuscript.

## Supplementary Material

Additional file 1: Table S1 Device-tissue interactions. Click here for file

Additional file 2: Table S2 Review of stapling devices and potential information required to address the existing surgical knowledge gap. Click here for file

Additional file 3: Table S3 Review of energy devices and potential information required to address the existing surgical knowledge gap. Click here for file

Additional file 4: Table S4 Review of access devices and potential information required to address the existing surgical knowledge gap. Click here for file

## References

[B1] Makino T Shukla PJ Samuels JD Rubino F Milsom JW Identifying specific surgical tools and methods for laparoscopic colorectal operations in obese patients J Gastrointest Surg 2012 16 2304 2311 10.1007/s11605-012-1937-z 22798184

[B2] Nakamura Y Kiaii B Chu MWA Minimally invasive surgical therapies for atrial fibrillation ISRN Cardiol 2012 2012 606324 22666609 10.5402/2012/606324 PMC3362139

[B3] Sandler BJ Horgan S Tichansky DS, Morton J, Jones DB Robotic surgical outcomes and safety The SAGES Manual of Quality, Outcomes and Patient Safety 2012 New York: Springer Science+Business Media, LLC 335 346

[B4] Msezane LP Katz MH Gofrit ON Shalhav AL Zorn KC Hemostatic agents and instruments in laparoscopic renal surgery J Endourol 2008 22 403 408 10.1089/end.2007.9844 18355135

[B5] Lacy AM Adelsdorfer C Delgado S Sylla P Rattner DW Minilaparoscopy-assisted transrectal low anterior resection (LAR): a preliminary study Surg Endosc 2013 27 339 346 10.1007/s00464-012-2443-9 22806513

[B6] Pai M Spalding D Jiao L Habib N Use of bipolar radiofrequency in parenchymal transection of the liver, pancreas and kidney Dig Surg 2012 29 43 47 10.1159/000335732 22441619

[B7] Feldman LS Fuchshuber P Jones DB Mischna J Schwaitzberg SD the FUSE (Fundamental Use of Surgical Energy™) Task Force Surgeons don't know what they don't know about the safe use of energy in surgery Surg Endosc 2012 26 2735 2739 10.1007/s00464-012-2263-y 22538677

[B8] Schwaitzberg SD Jones DB Don't get burned from lack of knowledge Ann Surg 2012 256 219 220 10.1097/SLA.0b013e318260260c 22750755

[B9] Klingler CH Remzi M Marberger M Janetschek G Haemostasis in laparoscopy Eur Urol 2006 50 948 956 discussion 956–957 10.1016/j.eururo.2006.01.058 16647188

[B10] Innovating to improve patient outcomes: exploring surgeon, device and tissue interaction. Program presented at: 2011 SAGES annual meeting and postgraduate course 2011 Texas: San Antonio

[B11] Baker RS Foote J Kemmeter P Brady R Vroegop T Serveld M The science of stapling and leaks Obes Surg 2004 14 1290 1298 10.1381/0960892042583888 15603641

