# Supplementary material for: Device-tissue interactions: a collaborative communications system
Source: Ann Surg Innov Res. 2013 Jul 29;7:10. doi: 10.1186/1750-1164-7-10 (PMC3737119; doi:10.1186/1750-1164-7-10)
Supplement: Additional file 1: Table S1 — Device-tissue interactions. [file 1750-1164-7-10-S1.doc]

Additional file 2: Table S2 Review of stapling devices and potential information required to address the existing surgical knowledge gap

| **Device tissue interactions** | | **Device group: stapling** | **Potential research questions** |
| --- | --- | --- | --- |
| **Device** | Unit:  “what it is” | Staple | What should a staple look like when the stapler is deployed? How does the staple shape affect outcomes? |
|  | Outcome:  “what it does” | Staple line | What does an optimal staple line look like? How many rows are “right”? |
|  | Embodiments:  “what performs it” | Gastrointestinal anastomotic staplers/skin staplers/linear staplers/circular staplers/ curved cutters/articulation linear cutters | How does performance vary from a stapler to a cutter in staple formation? Which staplers offer less risk of stricture formation? Which staplers/cutters offer less risk of anastomotic leakage? |
| **Tissue** | Properties:  “what can be affected” | Mechanical/biochemical | How do the components (air, liquid and solid) and nature of tissue affect staple formation? |
|  | Perfusion:  “what should not be affected” | Adequate/inadequate | How much blood flow is right for stapled tissue? How can we judge appropriate perfusion? Which tissues are most sensitive to ischemia? |
| **Interaction** | Dynamics:  “what is affected” | Compression/tension | What is the value of compression in stapling? How much tension is too much? How much of a load does tissue impart on devices? What is the staple line tensile strength after healing? Which stapler cartridges should I utilize for bronchial cartilage vs. the parenchyma? How do I minimize the trauma to the surrounding tissues? |
