# Supplementary material for: Device-tissue interactions: a collaborative communications system
Source: Ann Surg Innov Res. 2013 Jul 29;7:10. doi: 10.1186/1750-1164-7-10 (PMC3737119; doi:10.1186/1750-1164-7-10)
Supplement: Additional file 3: Table S3 — Review of energy devices and potential information required to address the existing surgical knowledge gap. [file 1750-1164-7-10-S3.doc]

Additional file 4: Table S4 Review of access devices and potential information required to address the existing surgical knowledge gap

| **DEVICE TISSUE INTERACTIONS** | | **Device group: access** | **Potential research questions** |
| --- | --- | --- | --- |
| **Device** | Unit:  “what it is” | Blade tip | How do different methods of entry affect outcomes? |
|  | Outcome:  “what it does” | Provides surgical access | How does size or location of defect impact outcomes? |
|  | Embodiments:  “what performs it” | Various trocars (blunt, optic/bladed), Microlap, Hassan/percutaneous instruments/video-assisted thorascopic surgery (VATS)/robotic-assisted procedures/HALS ports/single-port access surgery (SILS)/natural-orifice transluminal endoscopic surgery (NOTES) | How are the Hassan, blind entry, and Veress needle methods different in outcomes? How might outcomes change with the use of surgical robotics? Which access devices offer optimal oncologic resection with minimization of seeding? Which options offer fewer incisions, less pain, and shorter hospital durations? Under what circumstances can VATS be used vs. open procedures? When can a natural orifice be safely used and are the outcomes similar to other access devices? |
| **Tissue** | Properties:  “what can be affected” | Mechanical/biochemical | What are the different characteristics of the layers of tissue that a trocar has to go through/support? Which access devices and positioning offer less risk of herniation, perforation, bleeding or infection? How can access be maximized while allowing adequate perfusion of surrounding tissues? |
|  | Perfusion:  “what should not be affected” | Adequate/inadequate | How does perfusion relate to healing? What other supportive measures should take place during a procedure to maximize the perfusion of tissues? What is the best way to balance perfusion and hemostasis? |
| **Interaction** | Dynamics:  “what is affected” | Compression/stretching | How does trocar motion affect outcomes? How does the stretching of a small fascial defect accommodate a larger trocar impact healing? What are the effects of extending incisions in the middle of surgery? |
